# Identity processes and eating disorder symptoms during university adjustment: a cross-sectional study

**DOI:** 10.1186/s40337-021-00399-4

**Published:** 2021-04-09

**Authors:** Aoife-Marie Foran, Orla T. Muldoon, Aisling T. O’Donnell

**Affiliations:** Centre for Social Issues Research, Department of Psychology, University of Limerick, Castletroy, Limerick, Republic of Ireland

**Keywords:** Affiliative identity, Eating disorder symptoms, Social support, Injunctive norms

## Abstract

**Background:**

Young people with eating disorders (EDs) and ED symptoms are at risk during university adjustment, suggesting a need to protect their health. The social identity approach proposes that people’s social connections – and the identity-related behaviour they derive from them – are important for promoting positive health outcomes. However, there is a limited understanding as to how meaningful everyday connections, supported by affiliative identities, may act to reduce ED symptoms during a life transition.

**Methods:**

Two hundred eighty-one first year university students with an ED or ED symptoms completed an online survey during the first month of university. Participants completed self-reported measures of affiliative identity, social support, injunctive norms and ED symptoms. Path analysis was used to test a hypothesised mediated model, whereby affiliative identity has a significant indirect relation with ED symptoms via social support and injunctive norms.

**Results:**

Results support the hypothesised model. We show that affiliative identity predicts lower self-reported ED symptoms, because of its relation with social support and injunctive norms.

**Conclusions:**

The findings imply that affiliative identities have a positive impact on ED symptoms during university adjustment, because the social support derived from affiliative identity is associated with how people perceive norms around disordered eating. Our discussion emphasises the possibility of identity processes being a social cure for those at risk of ED symptoms.

## Plain English summary

Adjustment to university is a major challenge for many young people, as it often involves a change in meaningful connections. Young people with eating disorders (EDs) and ED symptoms are at risk during this transition. Research outside the ED field shows that people’s social connections can have important implications for health behaviours. This study aimed to explore how meaningful everyday connections may help reduce ED symptoms during university adjustment. Two hundred eighty-one first year university students with an ED or ED symptoms completed an online survey during the first month of university. Meaningful everyday connections were shown to have positive implications for ED symptoms during university adjustment. This is because the social support received from these connections is associated with how people perceive group norms around disordered eating. Health professionals working with people with EDs or at risk of an ED, may profit from this research as it speaks to the benefits of incorporating connections that are important to each person during periods of adjustment or life change.

### Identity processes and eating disorder symptoms during university adjustment: a cross-sectional study

Adjustment to university is a major challenge for young people. This is because for many, a transition to university involves a natural change in meaningful connections [1]. Therefore, it is not surprising that young people with disordered eating are at risk during this transition and may report compromised health outcomes [2]. Understanding how to manage such transitions may allow people with EDs and ED symptoms to protect their health. Speaking to this issue, the social identity approach suggests that people’s social connections – and the identity-related behaviour they derive from them – are important for improving health [3]. Researchers have also shown how identity continuity in times of transition is important for health outcomes [4, 5]. While much of the research in the ED literature has focused on how identities can maintain disordered behaviours, McNamara et al. demonstrated that a shared sense of identity with others in an online support group can reduce ED symptoms [6]. However, there is limited understanding of the role that other important identities may play. Thus, drawing on work in the ‘social cure’ tradition [3], we argue the importance of understanding how meaningful everyday connections, supported by affiliative identities, impact people’s ED symptoms in the face of a life transition.

The social identity approach, which incorporates social identity theory [7] and self-categorisation theory [8], offers a rich framework for understanding how identity processes might work for young people who are at risk of exacerbating ED symptoms. Tajfel defined social identity as the sense of self derived from meaningful connections [9]. Depending on the context, social identities can act as a benefit or a burden to people’s health [3]. To date, social identity researchers have focused their efforts on understanding how identification with therapy and support groups can influence ED symptoms [6, 10]. However, we argue different types of identity, such as identification with those who are an important part of our everyday lives, must also be considered to facilitate understanding of the relation between identity and ED symptoms. Billig’s conceptualisation of the banality has been used to refer to social identities that are unexpressed, but nevertheless present and available when required [11]. In other words, banal identities refers to “background identities” which are often unnoticed and taken for granted. They are linked to group memberships that are so inherent to who we are that they are often unremarkable, unworthy of comment, banal. These banal identities give rise to affiliative identities: connections made up of groups, such as family and friend networks, that are the assumed backdrop to everyday life [12]. These affiliative identities hinge on feelings of belongingness and while they may not be within our conscious awareness, they are ready to be mobilised during periods of adjustment.

Prior research suggests that pre-existing identities play an important role during the university adjustment period as they have shown to act as a basis for forming new identities [1]. Iyer and colleagues also found that people who have a history of successful meaningful connections are more likely to develop new connections [1]. In the context of this research, affiliative identities represent potentially important connections that have existed prior to university transition and should be present during the university adjustment period [4]. These can provide a sense of identity continuity to people with EDs and ED symptoms, which not only assists in a successful adjustment, but also has the potential to optimise their health. Thus, we argue affiliative identities are important for understanding ED symptoms as they can play an important role in adjusting to change [12], associated with university transition.

Affiliative identity makes social support possible, and thereby the receipt of support is implicated in the health-protecting benefits of identities [13]. The basic premise of the social identity approach is that belonging to a group provides people with a definition of who they are. If a person feels that being a group member is important to them, they are more likely to construe the support they receive more positively [14]. While there is strong evidence that social support is likely to be a critical mediator of the relation between identity and health [15], the role that this plays in promoting fewer ED symptoms during university adjustment remains to be tested. However, significant contributions have been offered suggesting that the perception of poor social support from meaningful connections can be considered as a risk factor for the development of ED symptoms [16]. Given that young people with EDs and ED symptoms are at significant risk of increases in ED behaviours during university adjustment [2], there is an imperative to understand how affiliative identities can provide support to protect their health.

Researchers in the social identity literature have also argued the importance of norms for understanding eating behaviour [17]. Norms are a broad concept with diverse meanings, and therefore researchers must consider the types of norms important for understanding specific behaviours. Descriptive norms examine what other group members do. Injunctive norms examine the perception of what group members endorse as appropriate and emerge through everyday connections. Both types of norms have shown to predict health-related outcomes [18]. However, we argue that injunctive norms are most important for ED symptoms, as they have shown to be useful for understanding social dimensions of eating behaviour [17]. Hence, the present research focuses on injunctive norms around disordered eating behaviours.

Prior research suggests that norms have an effect on eating behaviours. For instance, researchers have demonstrated that in a college environment, norms around healthy eating and exercise habits were associated with healthier eating behaviours [19]. Cruwys et al. also showed that normative change during an ED group program predicted improvements in dieting intentions and body dissatisfaction [18]. On the other hand, people who strongly identify as students, and perceived unhealthy eating as normative of student populations, were more likely to report unhealthy eating intentions [20]. Similarly, Neumark-Sztainer et al. showed the importance of dieting norms in determining the frequency of disordered eating in adolescents [21]. Based on these findings, it appears that the norms associated with a particular group have the potential to promote healthy or unhealthy eating behaviours. We propose that the impact of both affiliative identity and social support on ED symptoms may be contingent on whether injunctive norms promote healthy or disordered eating behaviours. Therefore, we argue the need to consider a combination of identity, social support and norms in order to generate a more accurate representation of the processes influencing ED symptoms.

Despite recognition of the importance of identity and social support for ED symptoms, at present the precise nature of these relations remain unclear. Researchers in the clinical ED field suggest that ED symptoms can be reduced through the support and understanding of people in therapy groups [6, 22]. Contrarily, ED groups may also contribute to the maintenance of disordered eating behaviours through identity-based support [23, 24]. While there have been numerous studies conducted which examine these variables separately, only a small number have considered how social support may underlie the relation between identity and ED symptoms. However, Leonidas et al. argued the need to move away from a reliance on ED therapy groups as a main source of support, to include significant connections, such as family and friends [25]. This is where the present research has particular value as we focus on social support derived from affiliative identities.

A body of evidence also points towards the interactive impact of identity and norms, such that people only conform to a norm when they strongly identify with its source [26]. For example, Stevenson et al. found that people are more likely to engage in certain eating behaviours, in part, because these behaviours are seen as normative of a group to which they affiliate [27]. Likewise, researchers in the addiction literature have shown that people who identified strongly with an addiction therapy group, whose norms endorsed lower substance use, showed greater abstinence behaviours [28, 29]. Given the associations between identity, norms and health behaviours, we argue that injunctive norms may also help explain the association between affiliative identity and ED symptoms.

Taking the available research together, it is evident that identity, social support and norms have important implications for ED symptoms. However, while studies have often examined the direct relations between these variables, no study as of yet has tested the interactive impact that these identity processes have on ED symptoms. Recent research by Cullum et al. showed when perceived social support derived from identification with peer groups is low, student drinking behaviours were strongly influenced by peer norms compared to when social support was high [30]. From these findings, Cullum and colleagues highlighted the benefits of considering the relation between perceived social support and norms for health behaviours [30]. And so, we argue that these feelings of social support drawn from affiliative groups may play a role in influencing people’s endorsement of disordered eating norms associated with the group. More specifically, we propose that affiliative identities can have benefits for ED symptoms because they afford opportunities to make meaningful contributions through the provision of social support and injunctive norms.

### The present study

The aim of the present research was to investigate and attempt to advance understanding of reciprocal processes between affiliative identity, social support, injunctive norms, and ED symptoms amongst a cohort of first year university students with an ED or concerns about ED symptoms. Given the challenges faced by young people with ED symptoms [2], it is important that we understand how they manage adjustment to university, so as to protect their health. The social identity approach has so far explored the role of therapy groups in ED symptomatology during periods of transition but has paid less attention as to how affiliative identities impact ED symptoms. Therefore, in light of previous research, we test a hypothetical model, which explores whether perceived social support and injunctive norms sequentially mediate the proposed relation between affiliative identity and ED symptoms.

## Methods

### Participants

First year students at an Irish university were recruited through email. Notably, participants were required to have concerns about ED symptoms or a diagnosed ED to take part. Two hundred eighty-one people (44 men and 237 women; *M*_*age*_ = 20.24, *SD* = 5.05, range 18–56 years) with either an ED or ED symptoms, took part in this study during the first month of their university transition. Twenty three people reported an ED diagnosis. These included anorexia nervosa (*n* = 14), bulimia nervosa (*n* = 5), binge-eating disorder (*n* = 3), muscle dysmorphia (*n* = 1), and other specified feeding or eating disorder (*n* = 2). Participants also indicated the types of disordered eating behaviours they engaged in: frequent dieting (*n* = 68); become anxious about specific foods (*n* = 102); meal skipping (*n* = 168); have a rigid routine surrounding exercise (*n* = 39); use exercise, food restriction, fasting or purging to make up for food consumed (*n* = 73); have a preoccupation with food, weight, and/or body image (*n* = 189); feel a loss of control around food (*n* = 122). Participants could choose more than one type of disordered eating behavior, if it applied to them. Only 36 out of the 281 participants reported seeking help for their concerns about disordered eating.

Based on the final sample size (*n* = 281), an alpha value of .05, and 80% power, sensitivity analysis using G*Power [31] indicated sensitivity to detect small effects (*f* = .028).

### Materials and procedure

The study had a cross-sectional and correlational design. Notably, the present study is part of a larger longitudinal study. Ethical approval was granted by the University of Limerick Faculty of Education and Health Sciences research ethics committee (ref: 2019_06_20_EHS). On receiving the recruitment email, potential participants were invited to click on the link provided to take part in online survey hosted using Qualtrics. The study complied with the ethical standards of the Declaration of Helsinki, including notifying participants that the study was voluntary, that they were free to withdraw at any time, and that they would indicate their informed consent by selecting the “agree to take part” button to start the survey. Participants first responded to demographic variables regarding their age, gender, type of disordered eating behaviours or ED diagnosis, duration of disordered eating concerns and whether help had been sought for these concerns.

### Predictor variable

#### Affiliative identity

Ashmore et al. argue the importance of self-categorisation and suggest that researchers should allow people to answer open-ended questions about their group memberships [32]. Consequently, self-categorised affiliative identity was measured in the present study with the question, “Which group of people you belong to is most important to who you are?” See Table 1 for a list of affiliative identities elicited from participants.

**Table 1:** Affiliative Identities Elicited from Participants

Family	155
School friends	108
College friends	3
Church friends	1
Hometown friends	1
Work friends	2
Partner/family	1
Sports team	4
Rugby team	2
Football team	1
Swim club	1
Scouts	2
Total	281

Identity strength, in relation to one’s most important identity, was measured using the four-item Group Identification Scale (Sani et al. [33]; e.g., I feel a bond with my [group], 1 = *strongly disagree,* 7 = *strongly agree,* Cronbach’s α = .92). This global scale examines one’s sense of belonging to the previously chosen group and one’s sense of commonality with in-group members. Each participant’s mean score on the four items was calculated, with higher mean scores equating to stronger affiliative identity.

### Mediator variables

#### Social support

The 19-item Medical Outcomes Study Social Support Survey (Sherbourne et al. [34]; e.g., Someone to listen to you when you need to talk, 1 = *none of the time*, 5 = *all of the time,* Cronbach’s α = .94) was used to measure perceived functional support. The questionnaire contains subsets of functional support including emotional/informational support, tangible support, affectionate support and positive social interaction. Items were averaged to compute mean scores, with higher mean score indicating greater perceived social support. This questionnaire is suited for application in the context of mental health disorders, as it is distinct from structural measures of social support (e.g. number of close friends).

#### Injunctive norms

Injunctive norms around disordered eating behaviours were established using four-items (Cronbach’s α = .70; adapted from Åstrosm et al. [35]; Smith et al. [36]; White et al. [37]: “Members of this group think that dieting is a good idea”(1 = *strongly disagree*, 7 = *strongly agree*), “If I were to lose weight, members of the group would …” (1 = *disapprove*, 7 = *approve*), “Members of this group think I …” (1 = *should not lose weight*, 7 = *should lose weight*), and “How many members of this group would think that being thin is a good thing?” (1 = *none of them*, 7 = *all of them*). Items were averaged to compute mean score, with higher score indicating greater perceived endorsement of disordered eating behaviours. This measure has also been used by Cruwys et al. [18] to examine injunctive norms in relation to eating behaviours.

### Outcome variable

#### Eating disorder symptoms

The 28-item Eating Disorder Examination Questionnaire (Fairburn et al. [38]; e.g., On how many of the past 28 days have you been deliberately trying to limit the amount of food you eat to influence your shape or weight, Cronbach’s α = .94) was utilised to measure self-reported ED symptomatology, including: restraint, weight concern, shape concern and eating concern. The 4 subscales capture the eating pathology spectrum, ranging from problems of undereating (i.e., anorexia nervosa) through to problems of overeating (i.e., binge-eating), with body dissatisfaction and bulimia nervosa falling in the middle. Participants were instructed that the questions were to be answered in relation to the 28 days prior to answering the survey. Items were averaged to compute mean scores, with higher mean scores indicating greater severity of ED symptoms.

### Approach to analysis

**Fig. 1:**
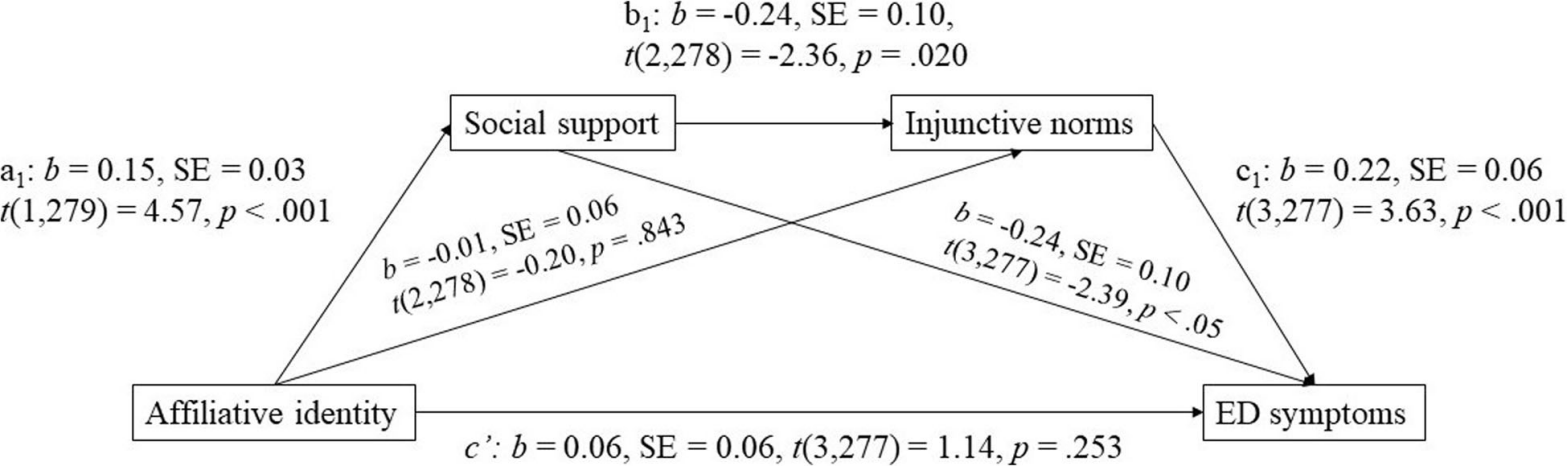
Serial mediation of the effect of affiliative identity on self-reported ED symptoms via social support and injunctive norms

All data were exported from Qualtrics to SPSS 25 for analysis. Preliminary analyses indicated that the variables of interest were not normally distributed. So, non-parametric tests were used for correlation analysis to evaluate the strength and direction of association between non normally distributed variables. First, we examined bivariate correlations between strength of affiliative identity, injunctive norms, social support, self-reported ED symptoms, age and gender (Table 2). Second, we used PROCESS serial mediation analysis (model 6) to test the relationship between affiliative identity (predictor variable) and self-reported ED symptoms (outcome variable) as well as the indirect effect of social support and injunctive norms (mediator variables). In Fig. 1, we illustrate this model. The effect of affiliative identity on ED symptoms is the product of the relation between affiliative identity and social support (labelled a_1_), social support and injunctive norms (labelled b_1_) and injunctive norms and ED symptoms (labelled c_1_). When examining indirect effects in this way, the mediators are hypothesized as a causal chain. However, given this was cross sectional data, following the suggestion of Hayes [39], we also tested alternative explanations that might fit the data (i.e. injunctive norms – social support – affiliative identity – ED symptoms). Age and gender were included in the model as covariates – as these correlated with self-reported ED symptoms in the preliminary analysis. The model was computed for each of the 10,000 bootstrapped re-samples, using bias-corrected 95% confidence intervals, and therefore can deal with non-normal distribution [39].

**Table 2:** Means, Standard Deviations and Bivariate Correlations of all Variables of Interest

	***M*** (***SD***)	1	2	3	4	Age	Gender
1. Affiliative identity	6.00 (1.35)	–	.41**	−.10	−.04		
2. Social support	3.81 (.79)		–	−.16**	−.11		
3. Injunctive norms	3.84 (1.28)			–	.21**		
4. ED symptoms	3.66 (1.34)				–	−.13*	.27**

*Note*: * *p* < .01; ** *p* < .001

## Results

### Indirect effect of Affiliative identity on ED symptoms

The serial mediation analysis conducted demonstrated that affiliative identity affects self-reported ED symptoms via perceived social support and injunctive norms (Fig. 1). As hypothesised, the results indicated that the effect of affiliative identity on self-reported ED symptoms was via perceived social support and injunctive norms, and though small, it was reliable with *b* = −.01, *SE* = .01, 95% CI [−.021, −.001]. The effect of affiliative identity on perceived social support was statistically significant, indicating that affiliative identity was associated with greater perceived social support. The effect of social support on injunctive norms, while controlling for affiliative identity, was also statistically significant, indicating that feelings of support were associated with lower perceived endorsement of disordered eating norms. Finally, the effect of norms on ED symptoms, while controlling for social support and affiliative identity, was also statistically significant. This indicated that those who perceived group disapproval of disordered eating report had lower ED symptoms. The relation between social support and ED symptoms, while controlling for affiliative identity, was significant, indicating that feelings of support were associated with lower ED symptoms. However, the relation between affiliative identity on injunctive norms was not significant. Consistent with our hypothesis that the relation between affiliative identity and self-reported ED symptoms is best understood with due attention to our mediators (social support and injunctive norms), there was no significant direct effect (see path labelled “c’” in Fig. 1). Overall, these findings indicate that affiliative identities have a positive impact on ED symptoms during university adjustment, because the social support linked to affiliative identity is associated with how people perceive norms around disordered eating.

Given that Cruwys et al. argue the predictive utility of norms for eating behaviours [40], a model of an alternative causal explanation (i.e., that injunctive norms had a significant indirect relation with self-reported ED symptoms via social support and affiliative identity as mediators) was also tested. This model was not significant: *b* = −.002, *SE* = .003, 95% CI [−.009, .002].

## Discussion

The present research explored the impact that identity processes have on ED symptoms, in a cohort of first year university students with a diagnosed ED or ED symptoms. In line with our hypothesis, we show that affiliative identity predicts lower self-reported ED symptoms, because of its relation with social support and injunctive norms. Consistent with the idea that social identity can be the basis of a social cure [3], we show that the social support derived from affiliative identity has positive implications for how people perceive norms around disordered eating, which subsequently predicts fewer self-reported ED symptoms. The present findings indicate that in the context of EDs, identity processes are not only important for understanding ED symptoms but may also predict fewer ED symptoms during periods of adjustment.

Notably, the present results do not indicate a direct relation between affiliative identity and self-reported ED symptoms. Instead, we show the relation between affiliative identity and ED symptoms, rests in a wider causal chain that includes perceived social support and injunctive norms. Research examining direct effects of social identity on ED symptoms have shown that identification with therapy groups can have both positive and negative implications for ED symptoms [10, 22]. This has parallels to social representations of use and misuse of substances, whereby dis-identification with a group oriented towards addictive behaviours alongside identification with an addiction therapy group can have implications for abstinence behaviours [28, 29]. However, Best et al. argued the extent to which social identities contribute to health behaviours depends on identity-related factors such as social support and the associated norms of the group [28] – highlighting the importance of examining indirect effects for gaining a greater understanding of how or why a relation occurs. In the present research, affiliative identity seems to be necessary but not sufficient for reducing ED symptoms. Our findings suggest that the association between affiliative identity, social support and injunctive norms, are identity processes that may play a role in reducing ED symptoms. Crucially, in examining this indirect effect, our research reveals an effect which would otherwise remain hidden.

The findings make an important contribution to our understanding of people with EDs and disordered eating who are at risk of exacerbating ED symptoms during university adjustment. The study shows that, as expected, injunctive norms are associated with ED symptoms [18, 19]. However, we again caution that this relation is associated with the extent to which people feel supported by their affiliative group. This is consistent with the hypothesis that belonging to a group (affiliative identity) makes social support possible [13, 15]. Our findings also support Cullum and colleagues’ suggestion that the level of social support one receives from their peer group may be an important factor in adherence to group norms around drinking [30]. In considering the indirect effect of social support and injunctive norms, the findings demonstrate how the interactive impact of these identity processes may act as a social cure for those at risk of ED symptoms. This supports recent qualitative findings by McNamara et al. which suggest that identification with online support groups can reduce ED behaviours because of the endorsement of positive group norms [6]. However, our findings imply that feelings of social support may have a positive impact on people’s perceptions of whether their affiliative group endorses norms around disordered eating, and thereby predicts lower ED symptoms. Therefore, we argue that meaningful everyday connections, supported by affiliative identities, have the potential to play a positive role in the health of people with EDs and ED symptoms during periods of adjustment.

### Implications

The present study represents an important addition to the literature within the social identity approach, as it shows how everyday identity processes matter for ED symptoms. We found that social support and injunctive norms are important explanatory factors in the relation between affiliative identity and ED symptoms. This indirect effect allows us to better understand the process underlying the relation between affiliative identity and ED symptoms, rather than a direct effect which would simply portray if affiliative identity predicted ED symptoms. As such, our findings provide evidence which may be used to improve interventions aimed at reducing ED symptoms. For example, group-based interventions that use affiliative identity as a platform for generating healthy norms around eating and access to functional social support, might be especially important to reducing ED symptoms. The present research offers an alternative conceptualisation of ED symptomatology as a social process, which is partially influenced by identity processes. Foran et al. argued the importance of examining social-psychological processes, which are likely to be significant in the context of ED psychopathology [41]. Our findings support this claim and show how moving away from individual level pathology offers prospects for group-based interventions that include identity processes that reduce ED symptomatology.

The present research also has important implications for health professionals working with people with EDs. First year college students are not only a high-risk population for ED symptoms, but are also at a life stage where sources of support are naturally changing in the transition to university, thus, increasing their risk. Given that the data were collected during the first 4 weeks of the transition to university, we argue our findings speak to the benefits of incorporating identities that are important to each person during periods of adjustment or life change. Indeed, family-based treatment has highlighted the important role of family in the treatment of children and adolescents with EDs. Our findings support the focus in treatment on group-based therapies that create a sense of belonging, to address a concern common to all members of the group. Importantly, the present study contributes to the ED field in suggesting that affiliative identities may be beneficial for ED symptoms during periods of adjustment because of the nature of these meaningful connections – through perceptions of support and injunctive norms.

### Limitations and future directions

The present research is not without limitations. The research design was cross-sectional and correlational. PROCESS Model 6 [39] was utilised in the present study as it was designed to establish the order of serial mediators in a causal pathway between predictor and outcome variables. Therefore, while the model cannot establish causation, in line with Hayes [39], we argue that this analysis approach does facilitate informed consideration of the potential causal pathways between the variables of interest. Also, as Cruwys et al. argue the predictive utility of group norms in understanding eating behaviours [40], we also explored an alternative model (i.e. that injunctive norms had a significant indirect relation with self-reported ED symptoms via social support and affiliative identity), which we found to be non-significant. While this approach cannot establish the direction of causal flow exactly, it rules out the possibility for an alternative causal order. Nevertheless, a longitudinal design should be employed to examine the role of identity processes on ED symptoms over time.

The present study also focuses on one type of health outcome important for people with EDs: ED symptomatology. This outcome was selected because to date, ED health outcomes have mainly been focused on physiological measures such as BMI and/or weight [42]. Research on identity processes that facilitate understanding of ED symptoms is important, as recent research suggests that the estimated prevalence of EDs is increasing [43]. Given that EDs are complex psychological disorders that affect every aspect of a person’s functioning, it is imperative that we consider a range of health outcomes in ED research. Social identity researchers have suggested a link between identity and a variety of psychological and social health outcomes, such as well-being and social functioning [3, 13]. Considering this, further research is needed to understand to what extent identity processes have implications for psychological and social functioning, in individuals with EDs and ED symptoms.

## Conclusion

Adjustment to university is a major challenge as it often involves a natural change in meaningful connections. Our findings show that affiliative identity predicts fewer self-reported ED symptoms because of its relation with social support and injunctive norms. We propose that affiliative identities may provide important psychological resources necessary for predicting fewer ED symptoms during the university adjustment period. This presents the possibility of identity processes being a social cure for those at risk of ED symptoms. In applying the social identity approach, our study offers an alternative approach to understanding ED symptoms, by framing these behaviours as a social process. Not only are our findings important for theoretical reasons but they also have the potential to inform practical interventions – in pointing to the importance of harnessing identity processes for ED health outcomes.

## Data Availability

The data generated and/or analysed during the current study are available on the Open Science Framework (see: https://osf.io/xuqa8/?view_only=e99d3638366047b4ba56af2e5dbbf841).

## Abbreviation

EDs: Eating disorders
